# The Diagnosis of Tumours

**Published:** 1899-04-08

**Authors:** T. Carwardine

**Affiliations:** Assistant Surgeon Bristol Royal Infirmary, Surgeon to the Orthopædic Hospital


### Hospital Clinics and Medical Progress.

### THE DIAGNOSIS OF TUMOURS.

Clinical Lecture by T. Carwardine, M.S., F.R.C.S., Assistant Surgeon Bristol Royal Infirmary, Surgeon to the Orthopædic Hospital.

We may take the word tumour in its general sense to mean a localised swelling, it may be of a portion of tissue or the whole of an organ. Mistakes are easily made in diagnosis. A son once made a mistake in diagnosis between pregnancy and ovarian cyst in a servant girl, and was reprimanded by his father, who soon afterwards made a like mistake and lost a titled patient. I have seen several patients supposed to have abdominal tumours, whose only malady was an excessive deposit of fat in the abdominal wall. *Phantom* tumours are often misleading, and are only dispelled under an anæsthetic. They usually consist of a tense bulging of the umbilical region through widely separated recti muscles in women who have borne many children. *Real* tumours may be cystic or solid, simple or malignant, the former being determined largely by the physical character and the latter by the general appreciation of the case in addition, founded on pathological knowledge and clinical experience. Some tumours can only be inferred from symptoms—*e.g.*, cerebral tumours.

In the diagnosis of tumours one should proceed methodically. At first a few questions are asked of the patient to gain confidence as well as knowledge of the complaint. One enquires as to when and why the tumour was first noticed, its character and rate of growth, and whether the growth was uniformly progressive or at any time diminished. If pain is associated with the tumour, then its distribution, duration and the causes which elicit it are determined. The position of the tumour will suggest other questions; for instance, in many abdominal tumours it will suggest the possibility of pregnancy, the nature of urination, the history of hæmaturia, jaundice, attacks of constipation, and the like. By this time the patient's confidence will have been gained, and the direct examination may proceed.

**Inspection.**—A tumour should always be scrutinised thoroughly before it is touched. The first point is to note the anatomical position. Thus an aneurism is in the line of a main artery, a renal tumour in one loin. The appearance of the skin may be reddened and shiny over an abscess, may have visible veins in the case of a nævus, nævo-lipoma, and over some new growths; it may be simply ulcerated, or invaded by new growths, or involved in fungating cysts. Then the general shape of the tumour is observed, for it is a rule that a tumour of an encapsuled organ tends to assume the shape of that organ. Any irregularity in the surface may be seen, such as pointing in the case of an abscess, bosses in a multilocular cyst.

**Palpation.**—This confirms and amplifies inspection. The outline of the tumour may be first mapped out with a warm hand, using the flat of the fingers and hand when examining the abdomen. The relations to the surrounding structures are next determined. Adhesion to skin is determined by gently pinching it up and finding it dimpled. Occurrence in a muscle or its sheath is detected by the patient putting the muscle into action, when the tumour will become fixed, whereas when above or below a muscle it may remain movable, the outline of the muscle being felt distinct from it, and their relations estimated. Then comes the question of the consistency of the tumour. An acute inflammatory swelling may give rise to a boggy and tender spot which is also soft to the touch. A tumour may be solid or cystic. If the latter the outline is usually uniform, and fluctuation, and perhaps a wave of percussion may be obtained. Fluctuation is a very important clinical sign, determined by pressing on the part with one hand and appreciating the wave-like sensation transmitted to the other hand held fixed. In large cysts the flat of the hand should be used. In very small collections one has to use the tips of the fingers. Fluctuation may be absent in the following conditions: When there is too little fluid to give the test, when the pus is not circumscribed, when the parts are very thick or non-pliant, and when the background is too yielding to afford any resistance. A percussion wave is usually obtained in large ovarian cysts and ascites by tapping on one side and receiving the impulse on the other with the flat of the opposite hand. A superficial wave around the parietes should be "damped" by placing something on the part to arrest the wave.

The edge of the tumour is an important part to carefully palpate. It may be lobulated: if in the subcutaneous fat and movable laterally this will be in favour of a lipoma; if deeply seated, and perhaps with small areas of fluctuation, the sign will indicate a multilocular cyst or multicystic tumour. If nodular, hard, and deeply seated, then the impression of malignancy will be conveyed. In a cyst the edge yields to the finger but does not shift away; the edge of a lipoma is firm to the finger and slips away under its pressure.

The uniformity of consistence determines whether or not the tumour be of a compound character, containing both cystic and solid elements, and this sign is of importance in the diagnosis or exclusion of malignancy according to situation.

Infiltration by a tumour is probably the most reliable of any single sign of malignancy. Malignant tumours are parasitic, and like all parasites grow at the expense of their host by incorporating their immediate surroundings. Accordingly there is usually an absence of definition of the margin, the tumour passes gradually into the healthy tissue, infiltrating it and giving rise to fixation, which is such a characteristic of malignancy. Indiscrimination may be spoken of as a sign of malignancy. Like the parasites of society the tumour grabs all it can get, and has little respect for the feelings of others. The chief exception to this is that malignant growths are very fond of veins, but dislike arteries. I have removed the whole internal jugular vein and part of the carotid sheath involved in a sarcomatous mass, which left the whole carotid artery quite free.

Tumours in connection with cavities may be reducible, and are then usually herniæ of some sort subcutaneously protruded through the parietes.

When a solid tumour is covered by fluid, as when an enlarged liver is associated with ascites, it may be felt only by the dipping test. In feeling the deep parts of a tumour in the abdomen one hand should be fixed flat upon the part, while the other hand presses upon the first.

**Percussion** in the diagnosis of tumours has already been referred to in the diagnosis of large cysts by the percussion-wave. Fluid and solid tumours have a dull note on percussion. Tumours containing gas yield notes of different intensity, pitch, and quality according to the position, size, and tension of the gas-containing tumour. The sense of resistance is of equal importance, and its appreciation is a faculty which the surgeon has to develop.

The shape and position of the dulness are of importance in the diagnosis of abdominal tumours. In ascites there is shifting dulness in the flanks and resonance above on account of the intestines floating on the surface of the fluid. In ovarian, bladder, and uterine tumours there is dulness above the pubes, having a convex upper margin, with resonance in the flanks. Tumours coming from above, such as an enlarged gall-bladder, leave the pubic and lumbar regions resonant, and have a convex lower margin.

**Auscultation.**—Simple auscultation detects peritoneal friction sounds, intestinal sounds, the fœtal heart, and various souffles. The "bell"-sound and auscultatory percussion are useful in defining the limits of a distended hollow viscus. The stethoscope is placed over the viscus, and the impact of two coins when over the same viscus gives rise to a bell sound, and percussion over the viscus has an easily distinguishable quality.

**Mensuration.**—Measurement is required when comparing the two sides of the body or two corresponding limbs, and for recording the rate of growth of a tumour. Abdominal measurements are sometimes of great value. In all cases of intestinal obstruction or of free gas in the abdominal cavity, the umbilical girth should be at once taken and registered on the temperature chart, for its gradual increase is often one of the surest signs of perforation. In enteric fever its rapid increase is often the only sign which draws attention to the fact of perforation. Tumours from the pelvis cause the distance from pubes to umbilicus to exceed that from umbilicus to the xiphoid cartilage, and tumours of the upper half of the abdomen tend to cause a converse condition. Tumours arising laterally, such, for instance, as an ovarian cyst, cause an increase from the anterior superior spine or from the costal margin of that side to the umbilicus.

The diagnosis of tumours is often a matter of extreme difficulty, but with the above principles in mind an accurate diagnosis is usually arrived at when supported by an extensive experience.

